# Guidelines on the diagnosis and management of the progressive ataxias

**DOI:** 10.1186/s13023-019-1013-9

**Published:** 2019-02-20

**Authors:** Rajith de Silva, Julie Greenfield, Arron Cook, Harriet Bonney, Julie Vallortigara, Barry Hunt, Paola Giunti

**Affiliations:** 1 Department of Neurology, Essex Centre for Neurological Sciences, Queen’s Hospital, Romford, RM7 0AG UK; 2 Ataxia UK, 12 Broadbent Close, London, N6 5JW UK; 3 Ataxia Centre, Department of Molecular Neurosciences, UCL Queen Sqaure Institute of Neurology, Queen Square, London, WC1N 3BG UK

## Abstract

The progressive ataxias are a group of rare and complicated neurological disorders, knowledge of which is often poor among healthcare professionals (HCPs). The patient support group Ataxia UK, recognising the lack of awareness of this group of conditions, has developed medical guidelines for the diagnosis and management of ataxia. Although ataxia can be a symptom of many common conditions, the focus here is on the progressive ataxias, and include hereditary ataxia (e.g. spinocerebellar ataxia (SCA), Friedreich’s ataxia (FRDA)), idiopathic sporadic cerebellar ataxia, and specific neurodegenerative disorders in which ataxia is the dominant symptom (e.g. cerebellar variant of multiple systems atrophy (MSA-C)). Over 100 different disorders can lead to ataxia, so diagnosis can be challenging. Although there are no disease-modifying treatments for most of these entities, many aspects of the conditions are treatable, and their identification by HCPs is vital. The early diagnosis and management of the (currently) few reversible causes are also of paramount importance. More than 30 UK health professionals with experience in the field contributed to the guidelines, their input reflecting their respective clinical expertise in various aspects of ataxia diagnosis and management. They reviewed the published literature in their fields, and provided summaries on “best” practice, including the grading of evidence available for interventions, using the Guideline International Network (GIN) criteria, in the relevant sections.

A Guideline Development Group, consisting of ataxia specialist neurologists and representatives of Ataxia UK (including patients and carers), reviewed all sections, produced recommendations with levels of evidence, and discussed modifications (where necessary) with contributors until consensus was reached. Where no specific published data existed, recommendations were based on data related to similar conditions (e.g. multiple sclerosis) and/or expert opinion. The guidelines aim to assist HCPs when caring for patients with progressive ataxia, indicate evidence-based (where it exists) and best practice, and act overall as a useful resource for clinicians involved in managing ataxic patients. They do, however, also highlight the urgent need to develop effective disease-modifying treatments, and, given the large number of recommendations based on “good practice points”, emphasise the need for further research to provide evidence for effective symptomatic therapies.

These guidelines are aimed predominantly at HCPs in secondary care (such as general neurologists, clinical geneticists, physiotherapists, speech and language therapists, occupational therapists, etc.) who provide care for individuals with progressive ataxia and their families, and not ataxia specialists. It is a useful, practical tool to forward to HCPs at the time referrals are made for on-going care, for example in the community.

**Electronic supplementary material:**

The online version of this article (10.1186/s13023-019-1013-9) contains supplementary material, which is available to authorized users.

## Introduction

The progressive ataxias are a heterogenous group of (individually) rare neurological conditions. Epidemiological evidence is lacking, but recent estimates suggest that there are at least 10,000 adults and 500 children with progressive ataxia in the UK [1, 2]. Whereas incidence rates for the progressive ataxias collectively are not known, some specific conditions have been well characterised. For example, Friedreich’s ataxia (FRDA), the most common inherited ataxia, has an estimated incidence rate of 1:29,000 amongst Caucasians [3].

The word ataxia means ‘lack of coordination’, and these conditions typically present with unsteadiness and imbalance, clumsiness, and slurred speech. Gait and balance problems often progress to the point at which patients become wheelchair-bound, and, in general, the level of disability progresses at the cost of functional independence. Communication becomes progressively impaired as a result of speech disturbances. Various other symptoms are associated with specific ataxia conditions, including spasticity, tremor, sensory disturbance, auditory and visual impairment, bladder and bowel dysfunction, cardiac complications, musculoskeletal complications, and cognitive impairment.

These rare and complex conditions present a significant diagnostic challenge, and both patients and clinicians alike have reported inefficient and arduous journeys which often fail to establish a definitive cause [4]. Beyond diagnosis, understanding of management options amongst HCPs is lacking, and as such patients face enormous challenges in both understanding their illness and obtaining treatment. Despite the absence of disease-modifying treatments for most ataxias, many aspects of these disorders are *treatable*, and it is essential that the responsible HCPs know how best to manage these symptoms optimally. The aim of these guidelines is therefore to increase awareness of these conditions among non-specialists HCPs (mostly in secondary care, such as general neurologists, clinical geneticists, physiotherapists, speech and language therapists, occupational therapists, etc.), and to improve their diagnosis and management. Recently the guidelines were affirmed and considered ready for dissemination by the European Reference Network for Rare Neurological Diseases, highlighting their recognition internationally.

These guidelines focus on the progressive ataxias, and exclude disorders where ataxia is an epiphenomenon of another neurological condition. Specifically, the recommendations cover the inherited ataxias (e.g. FRDA, SCAs), idiopathic sporadic cerebellar ataxia and specific neurological conditions in which ataxia is the dominant symptom (e.g. MSA-C). Of note, these guidelines do not cover ataxia that results from vascular, inflammatory or neoplastic pathology. In addition, information about the extra-neurological features of Ataxia Telangiectasia are not included in these guidelines, but these are covered elsewhere [5].

## Methods

The guidelines have been developed under the aegis of the patient support organisation, Ataxia UK, through extensive consultation with numerous UK neurologists, and other specialist physicians, surgeons, and therapists with experience in the diagnosis and management of ataxia. Contributors for each section were selected on account of their clinical expertise in aspects of ataxia diagnosis and management. More than 30 UK health professionals (please see Additional file 1: Table S1) contributed. They reviewed the available medical literature for their section using standard databases (e.g. PubMed, MEDLINE, Cochrane Database of Systematic Reviews, EMBASE and Scopus) and provided scientific evidence for the efficacy of different interventions. They graded the level of evidence following the Guideline International Network (GIN) protocol [6]. This included contributors critically reviewing the scientific evidence for the efficacy of interventions. For each paper, a judgement was made on the level of evidence (I to IV), any uncertainty was documented, and pertinent conclusions were drawn.

Earlier versions of the Ataxia UK guidelines (in 2007 and 2009) did not attempt to grade the quality of evidence supporting recommendations. In this iteration, data on the level of available evidence were used to allocate a *grade* to each recommendation, in accordance with criteria used internationally [7, 8]. Table 1 details the level of evidence and scheme for grading recommendations in these guidelines.

**Table 1:** Evidence grading scheme for these guidelines

Level of evidence [7]	
I	Evidence obtained from a systematic review of all relevant randomised controlled trials.
II	Evidence obtained from at least one randomised controlled trial.
III-a	Evidence obtained from one or more controlled trials, pseudo-randomised by alternate allocation, birth date or other planned method.
III-b	Evidence obtained from prospective or retrospective cohort studies with concurrent controls, case-control studies, or interrupted time-series with a control group.
III-c	Evidence obtained from cohort studies with historical controls, two or more single-arm studies, or interrupted time-series without a parallel control group.
IV	Evidence comprises opinions based on clinical experience, descriptive studies or reports by clinical bodies or committees.
Grading of recommendations [8]	
A	Body of evidence can be trusted to guide practice; includes one or more level I studies, or several at level II directly applicable to the target population, and demonstrating overall consistency of results.
B	Body of evidence can be trusted to guide practice in most situations; includes one or two studies rated as level II or several level III studies, directly applicable to the target population, and demonstrating overall consistency of results.
C	Body of evidence provides some support for recommendation(s) but care should be taken in its application; includes studies rated as III-c, or level I or II with a moderate risk of bias, some inconsistency and applicable to target population with caveats. Population studied is not the target population, however, it would make sense clinically to apply this evidence to target population.
D	Body of evidence is weak and recommendation must be applied with caution; includes level IV, or level I to IV studies with high risk of bias, inconsistent evidence and that are not applicable to target population.
GPP	Good practice point: Recommended best practice based on clinical experience and expert opinion.

A Guideline Development Group consisting of neurologists with expertise in ataxia and representatives of Ataxia UK reviewed all the sections and discussed any changes with contributors consensually.

## Results

The guidelines comprise 128 recommendations, grouped in four sections- on diagnosis and medical interventions, with two additional sections (available here in the Additional file 1) relating to in depth information about holistic/multi-disciplinary therapy and palliative care. Grading of the 128 recommendations were as follows: 6 graded B, 7 graded C, 10 graded D, and 105 graded as GPP. The full guidelines are available on the Ataxia UK website (https://www.ataxia.org.uk/Pages/Category/medical-guidelines).

### Diagnosis

The ataxias can present in a variety of ways, so an accurate and comprehensive history, together with clinical examination and relevant investigations are essential for efficient diagnosis and management. Important considerations in the history include the speed of symptom evolution, age at onset and family history. Patients with ataxia will typically report incoordination and unsteadiness, clumsiness, and slurred speech, and clinical signs include gait ataxia, nystagmus, hyper/hypometropic saccades and jerky pursuit when eye movements are assessed, slurred speech, intention tremor, dysmetria (or ‘past-pointing’), and dysdiadochokinesis. Diagnostic investigations are numerous and range from simple blood tests to next generation sequencing (NGS) panels, nerve conduction studies, lumbar puncture, and neuroimaging. Detailed information on tests that can be completed in primary and secondary care are shown in Table 2.

**Table 2:** Diagnostic investigations in adults

Primary care	U&Es Creatinine FBC ESR/CRP	Liver enzymes γ-GT TFT Vitamin B12	Folate Glucose CXR
Secondary care (first line)	αFP Blood film Caeruloplasmin/copper Coeliac screen Creatine kinase Genetic tests for FRDA, SCA 1, 2, 3, 6, 7 (12, 17) and FXTAS	Lactate Lipid-adjusted vitamin E and lipoproteins Lumbar puncture (cells, protein, glucose, cytology, oligoclonal bands, lactate, ferritin) MRI brain and cervical spine	Anti-Hu/Yo and other paraneoplastic antibodies Anti-GAD Anti-VGCC CT (chest, abdomen, pelvis) 14–3-3 and other proteins in CSF (prion diseases)
Secondary care (second line)	Cholestanol Plasma oxysterols Bile acids Coenzyme Q_10_(ubiquinone) Electroencephalography Very long chain fatty acids	Muscle biopsy Ophthalmology/OCT Peripheral nerve conduction studies Phytanic acid	Remaining genetic tests (NGS) Total body PET scan White cell enzymes

*U&Es* Urea and Electrolytes, *FBC* Full Blood Count, *ESR* Erythrocyte Sedimentation Rate, *CRP* C-Reactive Protein, *γ-GT* gamma-Glutamyltransferase, *TFT* Thyroid Function Test, *CXR* Chest X-ray, *αFP* alpha-Fetoprotein, *GAD* Glutamic Acid Decarboxylase, *VGCC* Voltage-gated Calcium Channel, *CT* Computerised Tomography, *FRDA* Friedreich’s Ataxia, *SCA* Spinocerebellar Ataxia, *FXTAS* Fragile X Tremor-Ataxia Syndrome, *CSF* Cerebrospinal Fluid, *OCT* Optical coherence tomography, *NGS* Next Generation Sequencing, *PET* Positron Emission Tomography

Rating scales to document the degree of impairment associated with ataxia are available, both generic (e.g. Scale for the Assessment and Rating of Ataxa (SARA), International Cooperative Ataxia Rating Scale (ICARS)) and specific for individuals forms of ataxia (e.g. Friedreich’s Ataxia Rating Scale (FARS)). Monitoring of ataxic patients’ impairments with one or more of these scales facilitates the objective recording of progression with time and enables the unbiased appraisal of interventions that are carried out. These measurements are frequently used in clinical trials of disease-modifying therapies, and in time are likely to become mandatory when such therapies are utilised in daily clinical practice (to monitor objectively the impact of such therapies on individual patients).

Table 3 summarises the main recommendations in this section.

**Table 3:** Diagnosis

Recommendations	Grade
The clinical context (speed of evolution, episodic/fluctuating versus progressive etc.) should determine the investigation of individual cases.	GPP
Ataxia in adults can arise due to serious neurological disease and urgent referral for secondary care (to a neurologist) should be made without delay following primary care investigation.	GPP
Children presenting with ataxic symptoms should be referred urgently for paediatric assessment (usually by local specialists, who may liaise with paediatric neurologists, clinical geneticists, etc).	GPP
Rapid progression (over weeks or months) can denote a paraneoplastic cause, prion disease or multiple system atrophy, thus urgent investigations are required.	GPP
When a diagnosis of progressive ataxia is made referral to a Specialist Ataxia Centre is encouraged.	GPP
Neurologists should liaise with their clinical genetics counterparts given the potential implication for family members of patients who undergo genetic testing.	GPP
Informed consent should be sought from all those undergoing genetic testing.	GPP
It is essential to offer genetic counselling to patients and discuss the implications of a genetic test prior to testing.	GPP
Genetic counselling should include the implications of having a genetic test for the individual and their family and any reproductive choices they may make.	GPP
Asymptomatic ‘at risk’ subjects should be offered genetic counselling.	GPP
Genetic testing of asymptomatic ‘at-risk’ minors is not generally recommended, but should be considered on a case-by-case basis.	GPP
Any genetic test results from research studies need to be validated by an accredited laboratory before a formal result is given to the patient.	GPP

### Medical interventions

As indicated earlier, many of the interventions to ameliorate the accompanying (usually neurological) complications in patients with ataxia are extrapolated from other conditions. The principles governing the management of spasticity and bladder symptoms could, for example, be equally well apply to patients with multiple sclerosis. In Table 4, key recommendations pertaining to the management of symptoms manifesting in ataxic patients are presented. The recommendations also cover some specific hereditary causes of ataxia, such as Episodic Ataxia type 2- where symptomatic therapies have the capacity to reduce the severity of, if not abort, bouts of ataxia.

**Table 4:** Symptomatic treatments

4.1 Spasticity	
Recommendation	Grade
Careful assessment by a neurologist, with advice from a physiotherapist, is required to decide on the type of treatment of spasticity.	GPP
Consider physiotherapy first to treat spasticity, and if that does not provide complete benefit use pharmacological treatment. Surgery should be considered in cases where physiotherapy and pharmacological treatments have not worked.	GPP
For pharmacological treatment of generalised spasticity consider using the following oral medications (usually in this order due to the profile of side effects and better tolerability): baclofen, tizanidine, gabapentin, clonazepam, dantrolene sodium or diazepam.	GPP
To treat focal spasticity refer to a specialised clinic for treatment with intramuscular botulinum toxin injections, followed by physiotherapy.	GPP
4.2 Tremor	
Recommendation	Grade
Patients with ataxia who have tremors should be offered pharmacological treatment using Propranolol, Primidone, Propranolol and Primidone in combination, Topiramate, Clonazepam and Gabapentin (in this order).	GPP
In patients where tremor is extremely debilitating and not responsive to medication a referral to a centre specialising in functional neurosurgery should be considered.	D [15–18]
4.3 Dystonia	
Recommendation	Grade
Focal dystonia should be treated with botulinum toxin injections.	GPP
Generalised dystonia should be treated with oral medications, followed by surgery if this is not effective.	GPP
Patients with dystonic tremor should be offered physiotherapy and oral medications followed by surgery if the former are ineffective.	GPP
4.4 Scoliosis	
Recommendation	Grade
Regular surveillance of the development of scoliosis in FRDA patients (especially children) is recommended as it is important for it to be treated.	GPP
If scoliosis is detected, referral to a physiotherapist and spinal surgeon is recommended.	GPP
For mild scoliosis the patient should be kept under close observation and the spinal surgeon should consider treatment with bracing.	B [19–21]
For severe scoliosis consider surgery to straighten the spine.	B [22, 23]
Regular follow-up by a spinal surgeon is recommended after an operation on the spine.	B [22, 23]
4.5 Pain	
Recommendation	Grade
Treat pain with physiotherapy and/or pharmacological treatments.	GPP
Consider use of the following drugs to treat neuropathic pain: Amitriptyline, Nortriptyline, Carbamazipine, Pregabalin, Gabapentin and Duloxetine.	GPP
Consider referral to a pain management clinic if pain is severe or limiting daily activities.	GPP
4.6 Cardiac involvement in FRDA	
Recommendation	Grade
When FRDA is diagnosed a referral to a cardiologist is recommended for the early diagnosis of cardiac problems and the management of cardiac complications, where required.	GPP
Regular screening by a cardiologist is recommended in FRDA patients; once every two years before any cardiac disease is documented, and at least annually after manifesting features of asymptomatic cardiac disease.	GPP
Transthoracic Echocardiography and ECG should be used for the diagnosis and monitoring of the myocardial changes.	GPP
Holter monitoring should be undertaken to detect silent cardiac arrhythmias or the association of symptoms (such as palpitations, shortness of breath) with the underlying rhythm.	GPP
A cardiologist should consider pharmacological treatment (including the use of anticoagulants), and in some cases the implantation of pacing devices, in collaboration with the neurologist.	GPP
4.7 Bladder problems - lower urinary tract dysfunction	
Recommendation	Grade
In primary care, test for urinary tract infection and measure post-void residual (to exclude common causes of urgency and frequency). If these are normal, check for other common causes such as prostate enlargement.	GPP
Practical advice should be given about cutting down caffeine, fizzy drinks and alcohol, as well as information about timed voiding and bladder retraining whenever appropriate. The fluid intake should be individualized; a fluid intake of between 1 to 2 L a day is recommended (taking into consideration possible concurrent cardiac issues).	GPP
Advice on pelvic floor exercises should be given as it may be helpful especially when symptoms are mild.	GPP
Most individuals with overactive bladder symptoms will require antimuscarinic medications (such as tolterodine, oxybutynin, propiverine and solifenacin).	GPP
In patients with cardiac complications and/or cognitive problems caution is advised when using antimuscarinic medications.	GPP
In patients with cognitive problems, more selectively-acting antimuscarinic medications, such as trospium chloride or darifenacin should be considered.	GPP
In some instances, referral to an urologist is recommended eg: in cases of haematuria or suspicion of concomitant urological condition.	GPP
4.8 Gastroenterological problems	
Recommendation	Grade
Suggest changes in lifestyle (eg: diet, fluid and mobility assistance) for patients with constipation, followed by the use of laxatives or suppositories.	GPP
Consider referral for specialist assessment if patients have urgency and faecal incontinence.	GPP
4.9 Sexual dysfunction	
Recommendation	Grade
Consider discussing sexual function with male patients due to the potential for erectile dysfunction.	GPP
Treat erectile dysfunction where appropriate with phosphodiesterase-5 inhibitors. Treatment decisions should balance the needs of the person and the potential side effect of medications e.g., hypotension.	GPP
If patients have cardiac pathologies caution should be exercised when considering medication, and consultation with a cardiologist is recommended.	GPP
4.10 Swallowing and dysphagia	
Recommendation	Grade
If patients show symptoms of dysphagia a referral to a speech and language therapist should be made (see Additional file 1*:* Table S2).	GPP
If there is unintentional weight loss due to dysphagia consider the use of nutritional supplements and refer to a dietician.	GPP
If calorie intake cannot be maintained despite supplements, discuss the possibility of a percutaneous gastronomy (PEG) to provide secure feeding.	GPP
4.11 Sialorrhoea (excessive salivation)	
Recommendation	Grade
Sialorrhoea is normally associated with dysphagia, thus a referral to a speech and language therapist is recommended for assessment of swallow.	GPP
Treat sialorrhoea and thick secretions according to Bavikatte et al. 2012 [9] (and the full guidelines).	GPP
4.12 Audiology and hearing	
Recommendation	Grade
If a patient is experiencing hearing problems refer to Audiology services for a battery of hearing tests.	GPP
A hearing aid trial should be considered although it is often not suitable for this patient population.	GPP
A trial with an FM hearing device is recommended in cases of ataxia with Auditory Neuropathy Spectrum Disorder (ANSD).	C [24, 25]
Refer to hearing therapist or speech and language therapist for guidance on communication tactics.	GPP
For those who do not achieve any benefit from hearing aids, consider a referral to a cochlear implant centre.	D [26]
In specific cases (e.g. ANSD) a referral to a neuro-otologist should be considered.	GPP
4.13 Eye symptoms	
Recommendation	Grade
A referral to a neuro-ophthalmologist is recommended if ataxia patients have any eye symptoms.	GPP
If disabling nystagmus or oscillopsia is present treatment is recommended, often with either gabapentin or baclofen.	B [27–29]
Refer to an optometrist or neuro-ophthalmologist for restoration of single vision with prisms in cases of diplopia.	GPP
Patients with visual impairment should be offered low vision aids and the possibility of having their visual disability registered.	GPP
4.14 Cognition	
Recommendation	Grade
When cognitive impairment is suspected (even if mild) referral to a Neuropsychology department is recommended.	GPP
Cognitive rehabilitation is recommended for those patients with cognitive impairment.	C [30]
Characterising the course of the cognitive impairment is advisable in order to inform the likely prognosis.	GPP
	GPP
4.15 Depression and other psychiatric symptoms	
Recommendation	Grade
In many cases depression can be treated in primary care using medications, counselling or cognitive behavioural therapy.	GPP
In more severe or complex cases of depression and other psychiatric symptoms a referral to a psychiatrist/neuropsychiatrist in secondary care is recommended.	GPP
For adults consult NICE Guidelines for the treatment of depression in patients with a chronic physical disorder [10].	GPP
4.16 Inherited episodic ataxias	
Recommendation	Grade
Advise episodic ataxia patients on identification and avoidance of common triggers that may cause attacks such as stress, caffeine and alcohol consumption, and excessive physical exertion.	GPP
Acetozolamide is recommended as the first line drug in episodic ataxia types 1 and 2, although not all patients respond.	GPP
Patients taking acetazolamide should be advised to keep hydrated to prevent the development of renal calculi and should undergo annual ultrasound screening of the urinary tract.	D
Patients with a known hypersensitivity to sulponamides should be counselled at the start of treatment and need to be kept under surveillance.	GPP
Consider use of 4-aminopyridine on a named patient basis as second line drug in episodic ataxia type 2 if acetazolamide is not beneficial.	C [31]
In episodic ataxia type 1 consider use of carbamazepine, phenytoin or lamotrigine as second line treatment.	GPP

To view the full content of the Medical intervention section, please see (https://www.ataxia.org.uk/Pages/News/Category/medical-interventions).

### Treatable ataxias

A small number of conditions presenting with ataxia are amenable to treatment, so diagnosing these is of great importance. Table 5 lists the treatable ataxias in adults and children, and recommendations for their diagnosis and treatment. Table 6 lists recommendations for the treatment of ataxias diagnosed specifically in childhood.

**Table 5:** Treatable ataxias

5.1 Gluten ataxia	
Recommendation	Grade
It is recommended that patients with idiopathic cerebellar ataxia are tested for gluten sensitivity.	GPP
Consider testing for antibodies against TG6 (when possible) as a more sensitive test for gluten ataxia.	C [32, 33]
Ataxia patients with or without enteropathy who have serological evidence of gluten sensitivity should be advised to start a gluten-free diet without delay.	C [34]
Patients who are starting a gluten-free diet should be advised about strict adherence and given dietetic advice.	GPP
Close monitoring is recommended with six-monthly testing to ensure for elimination of antigliadin antibodies.	GPP
5.2 Ataxia with vitamin E deficiency	
Recommendation	Grade
Patients diagnosed with ataxia with vitamin E deficiency or abetalipoproteinemia should be treated with vitamin E supplements.	C [35, 36]
5.3 Ataxia with vitamin B12 deficiency	
Recommendation	Grade
Patients diagnosed with ataxia and Vitamin B12 deficiency should be treated with Vitamin B12.	GPP
5.4 Ataxia with CoQ10 (ubiquinone) deficiency	
Recommendation	Grade
Patients diagnosed with ataxia with CoQ10 deficiency should be treated with CoQ10 supplements.	D [37–39]
Consider treatment of patients diagnosed with AOA1 with CoQ10 supplementation.	D [40, 41]
5.5 Cerebrotendinous xanthomatosis	
Recommendation	Grade
Prompt diagnosis of cerebrotendinous xanthomatosis is advised in order to initiate treatment.	GPP
If cerebrotendinous xanthomatosis is diagnosed treatment with chenodeoxycholic acid is recommended.	B [42, 43]
5.6 Niemann-Pick type C (NPC)	
Recommendation	Grade
If NPC is suspected based on clinical investigations, perform diagnostic tests described above. Early diagnosis is important as it is a treatable condition.	GPP
If NPC is diagnosed refer promptly to a Specialist Centre for treatment and management.	GPP
Treatment with Miglustat is recommended in both adult and paediatric cases and is available in Specialist Centres.	B [44–48]

**Table 6:** Treatable causes in children

6.1 Glucose transporter 1 deficiency	
Recommendation	Grade
If Glut-1 DS is diagnosed treat with a ketogenic diet.	D [49, 50]
6.2 Hypobetalipoproteinaemia	
Recommendations	Grade
Consider management of the moderate form of hypobetalipoproteinemia by reducing the proportion of fat in the patient’s diet and vitamin E supplementation.	GPP
6.3 Hartnup disease	
Recommendation	Grade
Consider treating Hartnup disease with nicotinamide or tryptophan-rich diet, and advise patients on a high protein diet, sunlight protection and avoidance of photosensitizing drugs.	GPP
6.4 Biotinidase deficiency	
Recommendation	Grade
Treat patients diagnosed with biotinidase deficiency with biotin.	GPP
6.5 Pyruvate deficiency	
Recommendation	Grade
Consider treatment with thiamine, carnitine or lipoic acid and advising on a ketogenic diet.	GPP
6.6 Structural disorders	
Recommendation	Grade
If ataxia is due to structural causes a referral for neurosurgical treatment may be recommended.	GPP

### Allied health professional interventions

Interventions by allied health professionals play a crucial role in the management of people with progressive ataxias. Additional file 1: Table S2 in the supplementary data highlights the key recommendations in the fields of physiotherapy, speech and language therapy and occupational therapy, and more detailed recommendations are available at https://www.ataxia.org.uk/Pages/News/Category/allied-health-professional-interventions. This section of the guidelines is especially valuable when referrals are made outside of specialist ataxia centres and their multidisciplinary teams, when it is recommended that on-going, “maintenance” therapy is carried out by community teams.

### Palliative care

Palliative care is an important element of the holistic care of individuals living with incurable conditions and their families, and aims to prevent and relieve suffering by means of the early identification, comprehensive assessment and treatment of pain and other complications (encompassing the physical, psychosocial and spiritual domains) [11]. Where appropriate, palliative care also includes end-of-life planning and care. In the absence of available literature on palliative and end-of-life care in the ataxias specifically, these recommendations are derived in part from evidence of these interventions in other progressive neurodegenerative conditions [12]. The main recommendations are summarised in Additional file 1: Table S3 in the supplementary data, and available in more detail at https://www.ataxia.org.uk/palliative-care.

## Conclusions and further research

When summarising the grades of evidence available for the recommendations highlighted in this paper, the large majority are of “Good Practice Point” standard and as such are based on clinical experience and anecdotal evidence. It is disappointing that more research has not been undertaken to support the evidence base of these interventions in clinical practice. An example is the many promising pilot studies in Physiotherapy, which have not been taken forward to larger randomised controlled trials (RCTs). Similarly, there are numerous pharmacological therapies that are being explored in pilot trials that need further investigation in larger, appropriately controlled studies.

In an era of limited health funding, the absence of evidence (through lack of trials rather than trials failing to prove efficacy) may be used to argue that these interventions should not be made available to ataxic patients, through publicly funded healthcare providers, such as the National Health Service in the UK. The need for further research is thus of paramount importance.

It is also of interest to note the limitations of the grading system adopted in the guidelines. There are some recommendations that do not lend themselves to this methodology. As such, these have been graded as Good Practice Pointers (GPP), as the absence of robust evidence from RCTs did not enable their categorisation at a higher grade. An example of this is the recommendation that genetic tests are undertaken in accredited laboratories. Although the intention of this recommendation is for it to be strongly endorsed (as is self-evident), it may appear weak as it is graded “GPP”. In instances such as this, the recommendations given do not lend themselves naturally to this method of grading. Nevertheless, this was only true for a minority of recommendations made and generally limited to the section related to diagnosis and to some recommendations on referrals to specialists.

Despite advances in diagnostic testing, many patients with ataxia are still not given a specific diagnosis of the underlying cause of their ataxias. The availability of gene panels, and increasingly exome and whole genome sequencing, may offer greater opportunities for the accurate (genetic) characterisation of ataxic subjects and families. Whilst novel genes responsible for causing ataxia might emerge, Next Generation Sequencing also poses challenges, in deciphering the true role of genetic/genomic variants, and these technological advances need to be coupled with expert bioinformatics. The determination of the pathogenicity of “variants of unknown significance” (VUS) can be time-consuming and expensive. This has engendered an interest in “deep phenotyping”- where novel biomarkers and functional assays are utilised to assist the interpretation of genetic tests. The use of Optical Coherence Tomography (OCT) in supporting the diagnosis of Autosomal Recessive Spastic Ataxia of Charlevoix-Saguenay is a recent illustrative example [51]. This type of integrative studies is likely to prove critical in deciphering the output from NGS (including VUS) over the next decade.

The production of this third edition of the guidelines has identified persisting gaps in the management of patients with ataxia. There is an urgent and pressing need to develop agents that have a disease-modifying effect in the ataxias, a need which is shared by almost all neurodegenerative disorders. This may partly reflect the level of understanding and complexity of the biological processes underpinning these disorders, and contrasts with the promising interventions now in common use in neuroinflammatory disorders such as Multiple Sclerosis. In the more common ataxias, such as FRDA and some SCAs, much research has focused on the identification of therapeutic targets and indeed several treatment trials have taken place. Furthermore, databases have been created and natural history data are being collected by networks of researchers worldwide, and this effort is proving useful in the design and implementation of trials [13, 14]. Indeed, as a result of these developments, and the financial incentives being provided for the study of rare diseases generally, pharmaceutical and biotech companies are increasingly engaging in ataxia research and running trials often in collaboration with academic centres and patient groups, such as Ataxia UK. Given the potential advantages of speedy development, drug repurposing approaches are also being explored by ataxia researchers worldwide.

In addition to disease-modifying treatments, there are specific symptoms experienced by patients with ataxia (such as balance and coordination problems, tremor and fatigue) for which effective therapies are also lacking yet can cause significant disability. Ataxia UK has independently surveyed the opinions of 426 people with progressive ataxia, to identify what they perceived to be their most pressing medical needs. Interestingly, impairments in balance, coordination and speech were highlighted as the symptoms with greatest impact, as well as *fatigue*. The production of these guidelines has revealed considerable overlap between what are seen to be areas requiring further research by professionals, and symptoms requiring most amelioration identified by patients. More research is thus needed to identify novel therapies and interventions to influence these symptoms, and critically to evaluate their effectiveness.

In creating this comprehensive guidance, our aim has been to provide a useful tool for HCPs looking after patients with progressive ataxia. Any comments on the recommendations are welcomed by the Guidelines Development Group, in order to refine and improve these recommendations in future editions.

## Additional file

Additional file 1: **Table S1.** List of contributors. **Table S2.** Allied health professional interventions. **Table S3.** Palliative care. (DOCX 27 kb)
