# Supplementary material for: Guidelines on the diagnosis and management of the progressive ataxias
Source: Orphanet J Rare Dis. 2019 Feb 20;14:51. doi: 10.1186/s13023-019-1013-9 (PMC6381619; doi:10.1186/s13023-019-1013-9)
Supplement: Supplementary file 1 — Table S1. List of contributors. Table S2. Allied health professional interventions. Table S3. Palliative care. (DOCX 27 kb) [file 13023_2019_1013_MOESM1_ESM.docx]

**Additional file 1**

**Table s1: List of contributors**

1. **Dr Claire Bates,** Consultant in Palliative Medicine, Queen’s Hospital, Romford, Essex.
2. **Dr Peter Baxter**, Paediatric Neurologist, Sheffield Children’s NHS Foundation Trust, Sheffield.
3. **Dr Harriet Bonney**, Medically Retired Speciality Doctor in General Adult Psychiatry, and Chair of Ataxia UK.
4. **Dr Fion Bremner**, Consultant Ophthalmic Surgeon, University College Hospital, the Royal Free Hospital & the National Hospital for Neurology and Neurosurgery, London.
5. **Dr Lisa Bunn**, Lecturer in Physiotherapy, Plymouth University.
6. **Dr Maria Carrillo Perez-Tome**, Clinical and Research Fellow in Cardiomyopathy, University College London Hospitals.
7. **Dr Mark Chung***,* Clinical Scientist in Audiology, Addenbrooke’s Hospital, Cambridge.
8. **Professor Lisa Cipolotti**, Head of Neuropsychology Department, National Hospital for Neurology and Neurosurgery, London.
9. **Dr Rajith de Silva**, Consultant Neurologist, Queen’s Hospital, Romford, Essex.
10. **Dr Kate Duberley**, former PhD student at UCL Institute of Neurology, London.
11. **Dr John Ealing**, Consultant Neurologist, Salford Royal NHS Foundation Trust, Manchester.
12. **Dr Anton Emmanuel**, Senior Lecturer in Neuro-Gastroenterology at UCL and Consultant Gastroenterologist at University College Hospital and the National Hospital for Neurology and Neurosurgery, London.
13. **Professor Paola Giunti,** Consultant Neurologist, National Hospital for Neurology and Neurosurgery, London.
14. **Dr Julie Greenfield**, Research Projects Manager, Ataxia UK.
15. **Professor Marios Hadjivassilliou**, Consultant Neurologist, Sheffield Teaching Hospitals NHS Foundation Trust.
16. **Mr N. S. Harshavardhana**, Clinical Spinal Fellow, Royal National Orthopaedic Hospital, London.
17. **Ms Kate Hayward**, Occupational Therapist, National Hospital for Neurology and Neurosurgery London.
18. **Professor Christian Hendriksz**, Clinical Lead and Consultant in Transitional Metabolic Medicine, Salford Royal NHS Foundation Trust, Manchester (and Extraordinary Professor Paediatrics and Child Health, University of Pretoria).
19. **Dr Joshua Hersheson**, PhD student, UCL Institute of Neurology, London.
20. **Professor Rita Horvath**, Professor of Neurogenetics, Newcastle University.
21. **Ms Joanne Hurford**, Occupational Therapist, National Hospital for Neurology and Neurosurgery London.
22. **Dr Fatima Jaffer**, PhD student, UCL Institute of Neurology.
23. **Dr Cherry Kilbride**, Senior Lecturer in Physiotherapy, Brunel University, London.
24. **Dr Anja Lowit**, Reader in Speech and Language Therapy at the University of Strathclyde, Glasgow.
25. **Professor Jonathan Marsden**, Professor and Chair in Rehabilitation, Plymouth University.
26. **Professor Andrea Nemeth**, Consultant in Neurogenetics and Associate Professor, Oxford University Hospitals NHS Trust and University of Oxford.
27. **Mr Hilali Noordeen**, Consultant Spinal Surgeon, Royal National Orthopaedic Hospital, London.
28. **Dr Jalesh Panicker**, Consultant Neurologist in Uro-Neurology, National Hospital for Neurology and Neurosurgery, London.
29. **Dr Antonios Pantazis**, Consultant Cardiologist, The Royal Brompton and Harefield Hospitals, London.
30. **Dr Michael H Parkinson**, Consultant Neurologist, UCL Institute of Neurology, London.
31. **Ms Liz Redmond**, Neurogenetics Nurse, Ataxia Clinic at National Hospital for Neurology and Neurosurgery, London.
32. **Dr Kai Uus**, Reader in Audiology, Manchester University.

| **Table s2: Allied health professional interventions** |  |
| --- | --- |
|  |  |
| **Recommendation** | **Grade** |
| Referral to a full range of therapies including speech and language therapy (SLT), physiotherapy (PT) and occupational therapy (OT) should be made available to patients with ataxia. | GPP |
| **s2.1 Speech and language therapy** |  |
| **Recommendation** | **Grade** |
| If patients experience specific difficulties with either their communication and/or swallowing a referral to SLT is recommended. An open referral system should be in place where patients are able to access help from SLT as and when required. | GPP |
| It is important that speech and language therapists (SLTs) undertake a comprehensive assessment of each patient’s communication, which takes into consideration the impact of communication difficulties on the individual’s activities of daily living and life roles. | GPP |
| SLTs should be vigilant for any signs of cognitive and/or hearing difficulties in patients with ataxia that might impact on communication, and the management strategy should be modified accordingly. | GPP |
| In the absence of evidence-based guidance on the most effective treatment, the therapist will need to devise individualised treatment programmes for dysarthria, based on findings of a comprehensive assessment. | GPP |
| When speech intelligibility levels fall below 50% or when reduced intelligibility has a significant impact on functional communication, alternative and augmentative means of communication should be considered. | D ^[52]^ |
| A comprehensive case history should be taken by the therapist- including the identification of signs and symptoms of dysphagia, detailed current eating and drinking behavior, and individual dietary preferences. | GPP |
| An instrumental examination of swallowing is indicated when information gained from clinical examination is not sufficient to guide management of the presenting dysphagia. | GPP |
| A multidisciplinary approach is recommended to dysphagia management between the therapist and dietician, to ensure optimal nutrition and hydration, as well between the therapist and the physiotherapist/occupational therapist to ensure optimal feeding position and use of aids or adaptations *(see table 13 in the full document for dysphagia management techniques).* | GPP |
| Muscle strengthening exercises can be indicated, and if so they should specifically target underlying swallowing pathophysiology. | GPP |

| **Table s2: Allied health professional interventions *(continued)*** |  |
| --- | --- |
|  |  |
| **s2.2 Physiotherapy** |  |
| **Recommendation** | **Grade** |
| Patients with progressive ataxia should be referred to see a physiotherapist or neuro-physiotherapist at an early stage of the disease in order to establish strategies to maintain function (e.g. balance, upper limb coordination, posture) and prevent falls. | GPP |
| Consider the potential use of rehabilitation approaches and the specific interventions for gait/balance and upper limb tremor for patients with ataxia on a case by case basis. | GPP |
| Consider suggesting rehearsal of intended steps through eye movement alone, i.e. looking at foot target placement for each step, before negotiating a cluttered room, as it might improve performance and safety. | D ^[53, 54]^ |
| Consider the use of video-game based coordinative programme in children with ataxia who can walk unaided under PT supervision. | C ^[55]^ |
| The use of walking aids is recommended and should be assessed on a case by case basis. Light touch as a balance aid may be helpful for postural orientation and stability. | GPP |
| Upper extremity weight bearing during ambulation may lead to worsening of gait parameters. It is important, therefore, for people with ataxia to decrease their dependency on weight bearing through the upper limbs (for example, by not leaning on furniture to assist when walking). | GPP |
| Careful assessment is required when recommending walking aids to patients with dysmetria, dysdiadochokinesia and tremor. | GPP |
| People with ataxia should be encouraged to exercise as part of health promotion but ensure that risk factors and health and safety considerations are assessed. | GPP |
| In patients with Friedreich’s ataxia and cardiac complications, advice form a cardiologist should be sought before embarking on an exercise program. | GPP |
| Assess seating position and posture when advising on a wheelchair. | GPP |
| Physiotherapists should be aware of the spectrum of additional specific impairments that people with progressive ataxia may have, such as fatigue, which also need to be treated. | GPP |

| **Table s2: Allied health professional interventions *(continued)*** |  |
| --- | --- |
|  |  |
| **s2.3 Occupational therapy** |  |
| **Recommendation** | **Grade** |
| When it becomes increasingly difficult for people with ataxia to perform everyday activities referral to OT services is recommended. | GPP |
| OT assessment tools should measure the person’s occupational engagement and/or satisfaction with their performance of an activity. | GPP |
| When making an assessment for treatment and management, therapists should refer to general considerations for intervention in the full guidelines. | GPP |
| Following a complete OT assessment, when a list of main concerns has been considered and treatment goals prioritised, consult practical suggestions in this section for guidance. | GPP/D |
| Fatigue management should be considered as part of the OT assessment. | D |
| Provide information on fatigue and discuss strategies, using activity analysis to help people look at alternative ways of completing tasks in a more energy efficient way. | GPP |
| Therapists should be mindful of the psychological state of the person with ataxia and refer to counselling or cognitive behavioural therapy as appropriate, and/or consider that anxiety management may be required. | D |
| Consider the need for future assessments when occupational needs changes and how the patient can re-access both OT and other appropriate services. | GPP |

| **Table s3: Palliative care** |  |
| --- | --- |
|  |  |
| **Recommendation** | **Grade** |
| All healthcare professionals should ensure patients are aware that Advance Care Planning is an option, and in appropriate contexts and in a suitable time and place should advise patients to consider doing it. | GPP |
| All healthcare professionals should facilitate advance care planning and documentation of advance care directives in individuals with ataxia. | GPP |
| Documented Advanced Care Planning for individuals with ataxia should be regularly reviewed by the individual in conjunction with their treating clinicians. Review may be instigated by the individual or care provider, can be part of regular review or may be triggered by a change in circumstances. | GPP |
| Consider referring to a specialist palliative care team when an individual with ataxia has complex distressing symptoms, psychological, social or spiritual needs, and/or a need for end-of-life planning. | GPP |
| Ensure an individual identified as approaching the end-of-life stage and their family have open access to specialist palliative care services. | GPP |
| If needed offer specialist input in the last few days of life, and aftercare and bereavement support to their families. | GPP |
